# Exposure of Developing Male Rats to One or Multiple Noise Sessions and Different Housing Conditions: Hippocampal Thioredoxin Changes and Behavioral Alterations

**DOI:** 10.3389/fnbeh.2019.00182

**Published:** 2019-08-13

**Authors:** Sonia Jazmín Molina, Gustavo Ezequiel Buján, Monserrat Rodriguez Gonzalez, Francisco Capani, Maria Eugenia Gómez-Casati, Laura Ruth Guelman

**Affiliations:** ^1^Centro de Estudios Farmacológicos y Botánicos (CEFyBO, UBA-CONICET), Facultad de Medicina, Consejo Nacional de Investigaciones Científicas y Técnicas, Universidad de Buenos Aires, Buenos Aires, Argentina; ^2^Facultad de Medicina, Cátedra de Farmacología, Universidad de Buenos Aires, Buenos Aires, Argentina; ^3^Consejo Nacional de Investigaciones Científicas y Técnicas, Instituto de Investigaciones Cardiológicas (ININCA, UBA-CONICET), Facultad de Medicina, Universidad de Buenos Aires, Buenos Aires, Argentina; ^4^Instituto de Ciencias Biomédicas, Facultad de Ciencias de la Salud, Universidad Autónoma de Chile, Santiago de Chile, Chile; ^5^Facultad de Medicina, Instituto de Farmacología, Universidad de Buenos Aires, Buenos Aires, Argentina

**Keywords:** noise, thioredoxin, behavior, hippocampus, enriched environment

## Abstract

Exposure of developing rats to noise has shown to induce hippocampal-related behavioral alterations that were prevented after a week of housing in an enriched environment. However, neither the effect of repeated exposures nor its impact on key endogenous antioxidants had been studied yet. Thus, the aim of the present work was to reveal novel data about hippocampal oxidative state through the measurement of possible age-related differences in the levels of hippocampal thioredoxins in rats exposed to noise at different developmental ages and subjected to different schemes and housing conditions. In addition, the possibility that oxidative changes could underlie hippocampal-related behavioral changes was also analyzed. Developing male Wistar rats were exposed to noise for 2 h, either once or for 5 days. Upon weaning, some animals were transferred to an enriched cage for 1 week, whereas others were kept in standard cages. One week later, auditory and behavioral assessments, as well as measurement of hippocampal thioredoxin, were performed. Whereas no changes in the auditory function were observed, significant behavioral differences were found, that varied according to the age, scheme of exposure and housing condition. In addition, a significant increase in Trx-1 levels was found in all noise-exposed groups housed in standard cages. Housing animals in an enriched environment for 1 week was effective in preventing most of these changes. These findings suggest that animals become less susceptible to undergo behavioral alterations after repeated exposure to an environmental challenge, probably due to the ability of adaptation to an unfavorable condition. Moreover, it could be hypothesized that damage to younger individuals could be more easily prevented by a housing manipulation.

## Introduction

Data from the literature have shown that exposure to noise could be capable to induce damage to the auditory system (Frenzilli et al., 2004; Gourévitch et al., 2014) as well as to structures of different extra-auditory tissues, such as brain structures (prefrontal cortex and hippocampus), cardiac tissues or adrenal and thyroid glands (Trapanotto et al., 2004; Manikandan et al., 2006; Uran et al., 2010; Gannouni et al., 2013; Molina et al., 2016a; Miceli et al., 2018). However, whereas exposure to occupational noise seems to be one of the main causes of disabling hearing loss, limited data are available concerning the effects of noise exposure on everyday lives of the ordinary population (Kopke et al., 2007). Actually, people living in big cities should be aware that they might be involuntarily exposed to high levels of noise coming from different sources. The urban traffic, the use of noisy household appliances or the attendance to concerts venues and discotheques might be examples of some of the many health-threatening environments.

It is well known that several environmental challenges increase the production of reactive oxygen species (ROS) in different tissues, which may overwhelm the endogenous antioxidant defenses and trigger a disturbance in the redox homeostasis (Erkal et al., 2006; Halliwell, 2006). In particular, it has been reported that exposure to noise was able to induce changes in the cochlear oxidative state (Yamane et al., 1995; Yamasoba et al., 1998; Dehne et al., 2000; Yamashita et al., 2004; Fetoni et al., 2015). In addition, Ohlemiller et al. (1999) reported a significant increase in ROS cochlear levels 1 h after exposure to noise, even when the acoustic stimulus is no longer present and Tamura et al. (2012) found that oxidative stress might be induced in the Corti organ of the inner ear after noise exposure in a rodent animal model. Finally, Kurioka et al. (2014) reported an increase in mitochondrial ROS production and excitotoxicity in the cochlea of rats exposed to noise.

ROS are unstable molecular species that contain one or more unpaired electrons that make them highly reactive (Halliwell, 1992). Hydrogen peroxide (H_2_O_2_), superoxide anion (${\text{O}}_{2}{}^{•-}$) or hydroxyl radicals (OH^•^) are ROS that have the ability to damage cellular lipids, proteins and to mitochondrial and nuclear genome through oxidative mechanisms, leading to mutations and cellular death (Halliwell and Gutteridge, 1991; Halliwell, 1992, 2006; Harman, 1992; Uttara et al., 2009; Massaad and Klann, 2011; Hanschmann et al., 2013). Although these species are persistently generated during aerobic respiration as derivatives of redox reactions and considering that even low amounts are required to regulate certain signaling pathways, an imbalance between the production of ROS and the system of endogenous antioxidants (i.e., a disproportionate increase in ROS levels and/or excessive decrease in antioxidant enzymes activities) might lead to cell damage (Jones, 2006). In fact, although the classic definition of oxidative stress focuses on an imbalance between pro- and anti-oxidative molecules in a given structure, at present this definition has been approached to a new concept in which oxidative stress is defined as the disruption of normally occurring redox signaling events (Jones, 2006).

It should be highlighted that brain is more susceptible to oxidative damage when compared with other tissues for different reasons. First, it consumes higher oxygen amounts; second, it has more iron content; third, it has high levels of unsaturated fatty acids and finally, it has lower activities of antioxidant enzymes such as superoxide dismutase and catalase. The high vulnerability can be observed after hypoxia (Romero et al., 2015; Ten and Starkov, 2012), ionizing radiation exposure (Caceres et al., 2009, 2010) and different nervous system disorders (Chen et al., 2010; Ma et al., 2012). Of importance, it has been reported that an environmental threat such as noise was able to induce an oxidative imbalance in different tissues (Cassarino and Bennett, 1999; Sathyasaikumar et al., 2007; Samson et al., 2008; Uran et al., 2010, 2012; Massaad and Klann, 2011; Molina et al., 2016b). A study of Zheng and Ariizumi (2007) showed an increase in oxidative stress and a suppression of the immune function after noise exposure during 28 days, whereas Cheng et al. (2011) found that only 1 week of moderate noise was capable to induce oxidative stress in different structures of mice brain. Cui and Li (2013) reported an increase in brain oxidative stress, as well as alterations of spatial memory in adult animals exposed to noise. Finally, several behavioral and biochemical changes were found in extra-auditory tissues of noise-exposed animals, including impairment of hippocampal-dependent reference and working spatial memory as well as changes in hippocampal antioxidant enzymes activities (Manikandan et al., 2006; Rabat et al., 2006) and a decrease in the number of hippocampal neurons (Jáuregui-Huerta et al., 2011).

Thioredoxins (Trx) are part of an endogenous family of oxido-reductases, recognized as the major reductant among a variety of antioxidant enzymes (Lillig and Holmgren, 2007; Lillig et al., 2008; Romero et al., 2015). Even though the Trx family includes various proteins, the main Trx isoforms are the cytosolic Trx-1 and the mitochondrial Trx-2 (Lillig et al., 2008; Aon-Bertolino et al., 2011; Godoy et al., 2011). Trx-1 is a regulator of cellular functions that take place in response to redox signals and modulates various signaling pathways. Different literature data show an increase in Trx-1 when an oxidative imbalance is induced in different nervous areas of animals subjected to neonatal hypoxia (Romero et al., 2015), intended to maintain a reduced environment to protect cells and tissues from oxidative damage (Silva-Adaya et al., 2014). In addition, Cunningham et al. (2015) showed that Trx-1 overexpression extended lifespan of transgenic mice by protecting against oxidative stress and Wu et al. (2015) found that a treatment with Trx-1 siRNA induced behavioral deficits. Therefore, it could be hypothesized that under physiological conditions the balance between ROS generation and antioxidant activity is highly controlled. However, when an injury is going on, an activation of the endogenous antioxidant defense systems can primarily occur as an attempt to counteract the oxidative process. Nevertheless, the endogenous antioxidant system often can fail in restoring redox homeostasis and the defense activity might result insufficient to prevent damage.

Last, a non-pharmacological neuroprotective strategy, the enriched environment (Laviola et al., 2008) has shown to be an effective tool that could be protective against different central nervous system (CNS) injuries (Lores-Arnaiz et al., 2006). It consists of a cage larger than the standard, which contains different toys, ramps and wheels. Although we have reported that EE was able to prevent noise-induced behavioral alterations in PND28 animals exposed at PND7 and PND15 to noise for 2 h (Molina et al., 2016a), data in animals exposed for 5 days have not been obtained yet.

Unfortunately, data obtained from developing animals exposed to noise are very scarce in the literature. The results from our laboratory showed different behavioral, biochemical and histological alterations when immature rats were exposed to noise. In addition, housing in an enriched environment has demonstrated to be an effective neuroprotective tool when rats were exposed to noise for a single day (Molina et al., 2016a). However, a comparison between the effects of single or repeated exposures to noise, at different developmental ages and/or housing conditions, as well as a possible relationship with the hippocampal oxidative state, has not been made yet.

Thus, the main hypothesis was that hippocampal thioredoxins might be responsible, at least in part, of the behavioral changes induced in developing rats after exposure to noise. Therefore, the aim of the present work was to reveal novel data about hippocampal oxidative state through the measurement of possible age-related differences in the levels of hippocampal Trx-1 and Trx-2, the major members of the thioredoxin family of endogenous antioxidants, in animals exposed to noise at 7 and 15 days according to different schemes. In addition, the possibility that oxidative changes could underlie hippocampal-related behavioral changes was also analyzed. Finally, the impact of housing conditions on noise-induced changes was additionally evaluated. To discard hearing alterations, the auditory pathway function was assessed.

## Materials and Methods

### Animals

Healthy male and female albino Wistar rats were obtained from the animal facilities of the Biochemistry and Pharmacy School, University of Buenos Aires, Argentina. A total of 30 multiparous females and 10 males were used for mating procedures. Pregnant rats were isolated and left undisturbed until delivery. The day of birth was designated as postnatal day (PND) 0. In average, 10 pups per litter were delivered and only male rats (usually 4–6 per litter) were used for the different experimental procedures.

To prevent from litter effects, no more than one animal from each litter was used to measure each parameter.

After behavioral and auditory experiments at PND28, animals were euthanized under a CO_2_ chamber for final disposal. Those animals assigned to western blot experiments were sacrificed through guillotine decapitation, the brain was exposed and the hippocampus was subsequently dissected.

**Figure 1:**
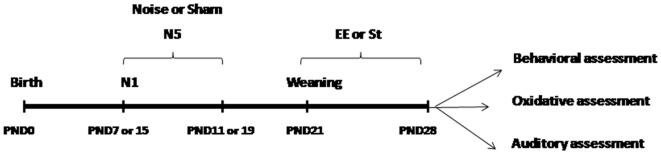
Experimental design. Sham: non-exposed animals; N1: single noise exposure; N5: five-daily noise exposure. St: standard housing. EE: enriched environment.

PND7 and PND15 littermates were randomly assigned to four experimental groups: sham (control) at PND7, sham (control) at PND15, noise-exposed at PND7 and noise-exposed at PND15 (*n* = 84 each group). In turn, within each group, animals received one of the following exposure schemes: single (N1) or five consecutive daily sessions (N5; *n* = 42 each group). Finally, each subgroup was divided into standard (St) or enriched (EE) cages housing, conforming 16 experimental groups (*n* = 21 each group). To reduce confounding factors, animals within each group were randomly assigned to the different measurements, being different those animals used for behavioral experiments (with some rats performing two behavioral tests, usually seven for OF and elevated plus maze (EPM) and other seven animals for IA) western blot experiments (four rats for each group) and auditory assessment (three rats for each group). Figure 1 depicts the experimental groups used.

All littermates were kept with their dams until weaning, at 21 days of age. Then, rats were separated and were put in groups of 2–3 in standard and 3–4 in enriched cage for 1 week with food and water *ad libitum*, on 12 h light-dark cycles (lights on at 7 A.M.) at 21 ± 2°C and mashed cornflower for bedding.

Animals were handled and sacrificed according to the Institutional Committee for the Use and Care of Laboratory Animal rules (CICUAL, School of Medicine, University of Buenos Aires, Argentina). The present experimental protocol was approved by this Committee and registered with the number 53679/16. The CICUAL adheres to the rules of the “Guide for the Care and Use of Laboratory Animals” (NIH; 2011 revision) and to the EC Directive 86/609/EEC (2010 revision) for animal experiments.

To avoid circadian rhythm alterations, noise exposures were performed in the intermediate phase of the light cycle, between 10 A.M. and 2 P.M. All experiments were performed in PND28 animals.

### Noise Exposure

For this procedure, the computer software TrueRTA was chosen to produce white noise, using a bandwidth from 20 Hz to 20,000 Hz in octave bands. For sound amplification, an active 2 way monitor (SKP, SK150A, 40 W RMS per channel) was used, located 30 cm above the animal cage, placed in an “*ad hoc*” wooden sound chamber of 1 m × 1 m × 1 m fitted with a ventilated top as reported by Cui et al. (2009). Before exposure, noise intensity was measured with an omnidirectional measurement condenser microphone (Behringer ECM 8000) by positioning the microphone in the sound chamber at several locations and taking an average of the different readings. Animals were kept in their home cages and the entire litter was assigned to the same group so that they were not handled throughout the exposure period. Sham animals were placed in the same chamber, but without being exposed to noise. Given that experimental animals were still being breastfed and mothers had to be transiently removed for the period in which the pups were being exposed to noise, this action was carried out also in non-exposed sham animals in order to discard possible changes that could be attributed to mother separation.

Based on previous publications of our laboratory (Uran et al., 2010) with further modifications (Molina et al., 2016a), PND7 and PND15 animals were exposed for 2 h to white noise at 95–97 dB SPL (20–20,000 Hz), either a single day (N1) or for five consecutive days (N5). Background noise level ranged between 50 and 55 dB SPL, being within the harmless interval suggested by the WHO guidelines (NIOSH, 1998) and by others (Campeau et al., 2002; Sasse et al., 2008). Lighting was provided by a 20 W lamp located in the upper left corner of the sound chamber. In addition, the chamber had a sound attenuation system made with Celotex^TM^.

The intensity and duration of noise used in the present work were chosen considering its potential translational value, as it could be comparable to the intensity and duration perceived in various workplaces, mainly induced by different machines, data that can be found even in the earliest WHO report (WHO, 1999).

### Enriched Environment (EE)

At weaning (PND21), a subset of animals was housed in an EE with 3–4 animals residing together whereas a subset of 2–3 was accommodated in standard cages (St). In contrast to St, conventional top-wired, stainless steel rectangular cages of 40 cm × 25 cm × 16 cm, EE consisted of 54 cm × 40 cm × 41 cm plastic cages with two levels, containing two connecting ramps. Different plastic toys and tunnels, as well as a running wheel, were placed in the cage. A palatable food, such as Froot Loops^®^, was added regularly in small quantities in addition to the conventional balanced food. It should be highlighted that the minimal sugar and fat amounts of the Froot Loops^®^ offered are much below those contained in a “cafeteria diet,” known to induce *per se* metabolic and behavioral changes (Zeeni et al., 2015). The objects were changed every 2 days to ensure continued novelty. Rats were maintained in their housing condition (St or EE) for 1 week, prior to behavioral studies.

### Auditory Pathway Assessment (ABR)

The auditory brainstem responses (ABRs) are sound-evoked potentials generated by neuronal circuits in the ascending auditory pathways and consequently require functional integrity of hair cells, as well as their afferent neurons.

PND28 animals were anesthetized with ketamine (100 mg/kg, i.p.) and xylazine (20 mg/kg, i.p.) and placed in an acoustically electrically shielded chamber maintained at 30°C. Methods for measuring ABRs were essentially as described (Kujawa and Liberman, 2009; Maison et al., 2013). Briefly, acoustic stimuli were delivered through an acoustic system consisting of two miniature dynamic earphones used as sound sources and an electret condenser microphone coupled to a probe tube to measure sound pressure near the eardrum. Digital stimulus generation and response processing were handled by digital I-O boards from National Instruments driven by custom software written in LabVIEW. ABRs were recorded with needle electrodes inserted at vertex and pinna with a ground reference near the tail. Auditory responses were evoked with 5 ms tone pips, amplified (10,000×), filtered to six different frequencies (0.1–3 kHz), and acquired on a computer. The sound level was raised in 10 dB steps and “threshold” was defined as the lowest SPL level at which a wave is detected.

To avoid potential data misinterpretation, animals assigned to ABR assessments were not subjected to further behavioral or biochemical evaluations and were euthanized in a CO_2_ chamber for final disposal.

### Behavioral Assessment

PND28 animals were used for all behavioral experiments. To control for variables that could significantly alter physiological and behavioral indicators of stress (Walf and Frye, 2007), animals remained in their home cage and placed in a separate area of the main housing room for 30 min prior to the behavioral assessments. Thereafter, they were individually housed for 5 min in the same area and finally were taken to the adjacent testing room, which had identical environmental conditions, for additional 3 min to complete the acclimation period, prior to the behavioral assessments.

#### Open Field Task (OF)

An open field device was used to analyze habituation memory and exploratory activity, behaviors known to depend on the hippocampus (Vianna et al., 2000; Barros et al., 2006). In this task, the reduction of locomotor activity triggered by a repeated exposure to the same environment can be taken as a measure of preservation of habituation memory (Vianna et al., 2000; Pereira et al., 2011). In addition, the activity in the first session of the OF can be used to assess changes in emotionality induced by exposure to a novel environment. In consequence, vertical exploratory activity can be quantified by recording the number of rearing and climbing, holding on the hind legs. The activity was recorded using a camcorder (Handycam DCR-DVD810, Sony).

- Apparatus: OF device consists of a 50 cm × 50 cm × 50 cm wooden box, with a floor divided into 25 equal squares by black lines.
- First session: rats were withdrawn from the cage, placed on the center rear quadrant of the OF box and allowed to freely explore the box for 5 min. The number of crossed lines as well as the number of rearing and climbing, were recorded over the session.
- Second session: after 1 h inter-trial in their home cages, animals were acclimatized to the behavioral room and allowed to explore the apparatus for another 5 min. The number of crossed lines was recorded and compared with the number crossed in the first session to evaluate habituation to the device (Barros et al., 2000).

#### Elevated Plus Maze (EPM)

This task was used to evaluate anxiety-related behaviors that depend on the integrity of the hippocampus (Montgomery, 1955; Brenes et al., 2009; Violle et al., 2009).

Anxiety-related behaviors are calculated as the number of entries to the open arms as well as the latency required to access the open arms. When an increase in the first and a decrease in the latter are observed, it could be stated that a decrease in anxiety-like behaviors could have occurred.

Additionally, some ethological parameters can be evaluated using this task (Carobrez and Bertoglio, 2005), designated as risk assessment behavior because they have been associated to detection and analysis of threats or threatening situations (Rodgers and Cole, 1993). One of these parameters is called head dipping (HD). As closed arms and center platform were designated as “protected” areas (i.e., offering relative security), the percentage of head-dipping in closed arms (%HD in closed arms) was calculated as the percentage of these behaviors displayed in or from the protected areas. Therefore, this parameter describes the action of the animal when it is positioned on a closed arm and, at the junction with the open arm, stretches the head over the ledge of an open arm and bends down.

- Apparatus: the wooden apparatus consists of four arms of equal dimensions (50 cm × 10 cm) and raises 50 cm above the floor. Two arms, enclosed by walls 40 cm high, are perpendicular to the two other opposed open arms.
- Session: rats were placed in one of the closed arms, facing the center of the maze, and were recorded for 5 min using a camcorder (Handycam DCR-DVD810, Sony). The number of entries to open arms, the latency to reach the open arms, as well as the percent of HD in closed arms, were calculated. Only few rats randomly distributed across experimental groups fell when they walked along the open arms; these animals were excluded from the study.

#### Inhibitory Avoidance Task (IA)

Inhibitory avoidance task was used to measure the memory of an aversive experience through the simple avoidance of a location in which the unpleasant experience occurred. This task is thought to depend heavily on the dorsal hippocampus and is a reliable index of associative memory (Ennaceur and Delacour, 1988; Izquierdo and Medina, 1997).

- Apparatus: the apparatus consists of a box (60 cm × 60 cm × 40 cm), divided into two compartments: one is illuminated while the other is equipped with a removable cover to allow it to be dark, as described by Roozendaal (2002). A removable partition divides the two compartments. The floor of the dark compartment consists of a stainless steel grid at the bottom, through which a continuous current could be delivered.
- Habituation session: the rat was placed into the lit box and allowed to freely explore the apparatus. Either after passing three times to the dark side or after 3 min staying in the dark side, the rat was removed from the apparatus. After 10 min, the rat was placed again in the lit side and when it entered the dark side, the doors were closed and the rat was retained for 10 s on this side.
- Training session (T1): after 1 h, each rat was placed in the lit compartment, facing away from the dark compartment; the latency to move into the dark compartment was recorded. When the rat stepped with all four paws in the dark compartment, a foot shock (1.2 mA, 2 s) was delivered. The rat was quickly removed from the apparatus and returned to its home cage.
- Retention session (T2): retention test was performed 1 h after the training session by following a similar procedure, except for the fact that no footshock was delivered. The ratio between the latency to move into the dark compartment in the *retention* and the *training* sessions (T2 and T1, respectively) was taken as a measure of associative memory retention (T2/T1).

### Western Blot Experiments

The levels of the Trx-1 and Trx-2 were determined in hippocampal homogenates of rats from all experimental groups through Western blot experiments. To prevent from confounding influences, those animals destined to western blot experiments were not previously used for behavioral or auditory measurements. Animals were euthanized through guillotine decapitation, brain was exposed, and hippocampus dissected. Briefly, tissues were homogenized in ice-cold lysis buffer (25 mM Hepes, 6 mM MgCl, 1 mM EDTA, mix of protease inhibitors) and centrifuged at 10,000 *g*. The supernatants were analyzed for total protein concentration using Bradford solution, with bovine serum albumin (BSA) as standard. According to the determined protein concentration, the samples were diluted with sample buffer solution (6×: 0.346 M SDS, 30% glycerol, 6% 2-mercaptoethanol, 0.179 mM bromophenol blue, 0.998 M Tris–HCl, pH 6.8) in order to have 10 μg of tissue/ml. Therefore, homogenates were preincubated with 1 μl DTT 1 M per 10 μl of sample for 30 min at room temperature and then heated to 94°C for 10 min. Then, samples were run on 14% polyacrylamide gels under denaturing conditions. The samples were electro-transferred to PVDF membranes which were blocked with 5% non-fat milk and 1% BSA and incubated overnight with the primary antibody at 4°C [Trx-1 and Trx-2 rabbit antibodies, used in a dilution of 1:1,000, were a generous gift of Dr. Lillig from University of Greifswald, Germany; sc-32233 GAPDH (load control) rabbit antibody from Santa Cruz Biotech. was used in a dilution of 1:5,000]. After that, samples were incubated at room temperature with the secondary anti-rabbit HRP-conjugated antibody (sc-2768 Santa Cruz Biotech., diluted 1:5,000) for 2 h under shaking, scanned densitometrically by the Image Quant analyzer and quantified using ImageJ software.

### Statistical Analysis

Normality test was performed for each group (KS-test). Significant differences between groups were analyzed through one-, two- or three-way analysis of variance (ANOVA) tests with LSD *post hoc* comparisons using the Infostat/L software. When the normality tests failed, a non-parametric analysis was made, using the Kruskal–Wallis test. Different letters (a, b, c, d) were used to depict significant differences between the means, being significantly different one bar from another when they have no common letters. For example, if a bar received an “a” score and another a “b” score, it means that they differ statistically with *p* < 0.05. Considering the large number of groups to be compared, with the consequent difficulties in data interpretation, the differences between the results of PND7 and PND15-exposed animals were analyzed separately. A probability < 0.05 was accepted as significant.

When interactions were significant, a simple effect analysis was performed, through which one-way ANOVA analyses were performed. The results were expressed as mean values ± standard error of the mean (SEM) and graphs were performed with Prism Graphpad software v5.

## Results

### Auditory Function

**Figure 2:**
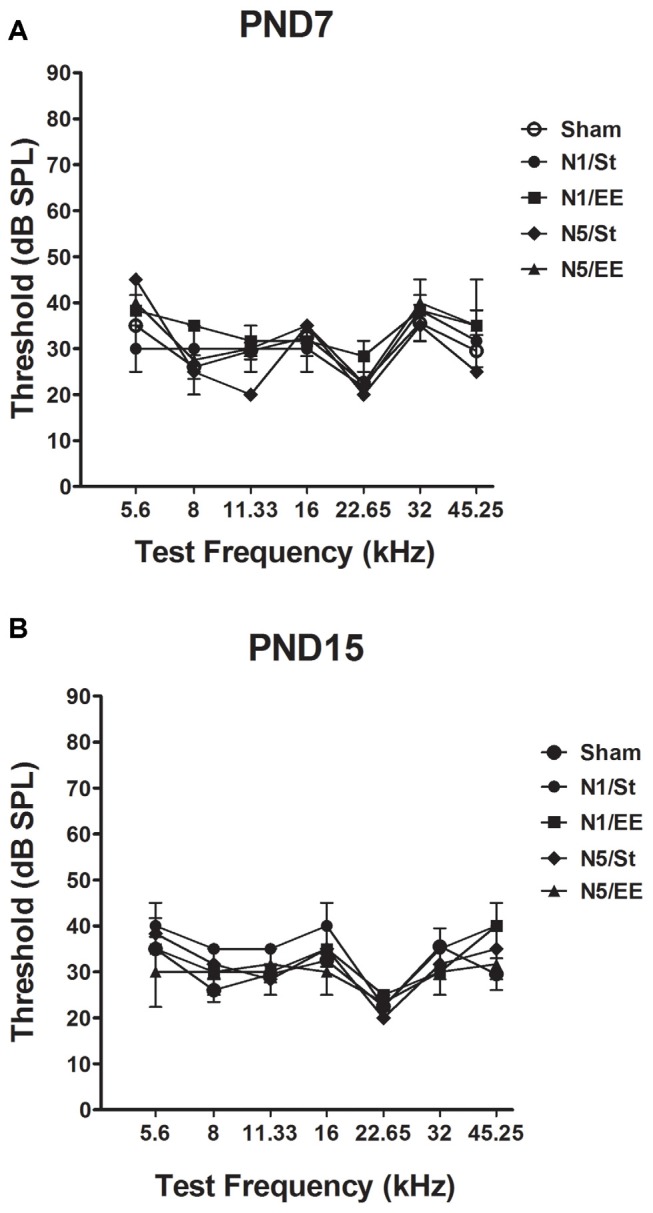
Auditory brainstem responses (ABRs) in PND28 animals exposed at **(A)** PND7 and **(B)** PND15. Sham: non- exposed animals; N1: single noise exposure; N5: five-daily noise exposure. St: standard housing. EE: enriched environment. Data represent the mean ± standard error of the mean (SEM) of the ABR, *n* = 3 for each group.

No significant changes in ABRs thresholds in any of the frequencies tested were observed in PND28 animals exposed to noise at PND7 and PND15 [non-parametric Kruskall–Wallis test, *H* < 4 and *p* > 0.05 (NS) for all frequencies, Figures 2A,B].

### Open Field (OF) Task

(i) The number of lines crossed in two sessions of 5 min in an OF, separated by an interval of 1 h, was taken as an index of short-term habituation to a new environment.

**Figure 3:**
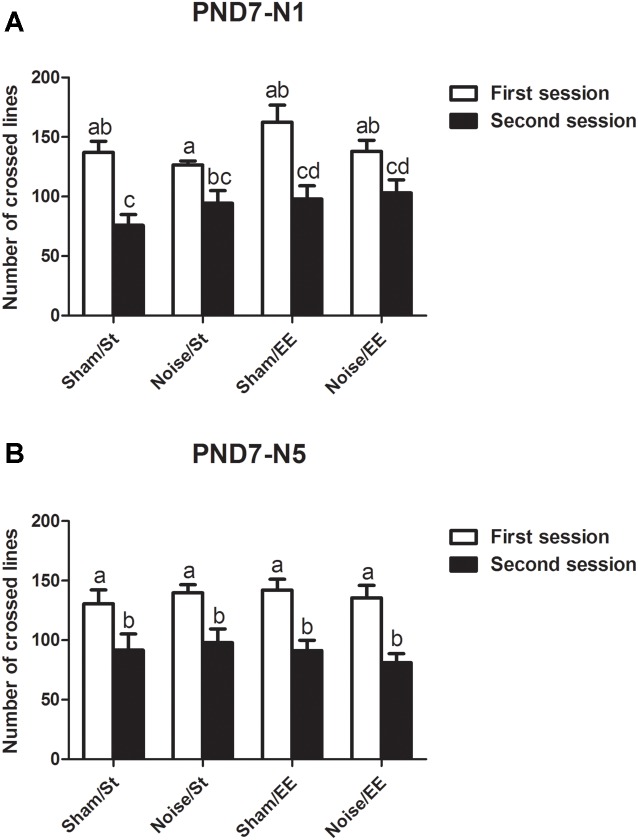
Number of lines crossed in the first and second session of the OF by PND28 animals exposed to noise at PND7. **(A)** PND7 animals exposed according to N1; **(B)** PND7 animals exposed according to N5. Sham: non- exposed animals; N1: single noise exposure; N5: five-daily noise exposure. St: standard housing. EE: enriched environment. Different letters (a, b, c, d) symbolize significant differences with *p* < 0.05. Data represent the mean ± SEM of the number of lines crossed in the first and second session of the OF, *n* = 7 for each group.

Data show that exposure to noise at PND7, according to N1 and N5 schemes, induced a decrease in the number of lines crossed in the second session of the OF when compared with the first session, both in standard or in enriched conditions, that resulted similar to what was observed in sham animals, when evaluated at PND28 [Figure 3A, *N1*: Three-way ANOVA, *F*_(7,65)_ = 7.77, *p* < 0.01. Between factors: exposure (sham or noise), *F*_(1,65)_ = 0.22, NS; housing (St or EE), *F*_(1,65)_ = 5.4, *p* < 0.05; within factor: session (first or second), *F*_(1,65)_ = 43.88, *p* < 0.01. *post hoc* comparisons: first vs. second session: all groups, *p* < 0.01. Figure 3B, *N5*: Three-way ANOVA, *F*_(7,67)_ = 6.29, *p* < 0.01. Between factors: exposure (sham or noise), *F*_(1,67)_ = 0.04, NS; housing (St or EE), *F*_(1.67)_ = 0.23, NS; within factor: session (first or second) *F*_(1,67)_ = 41.79, *p* < 0.01. *Post hoc* comparisons: first vs. second session: all groups, *p* < 0.05].

**Figure 4:**
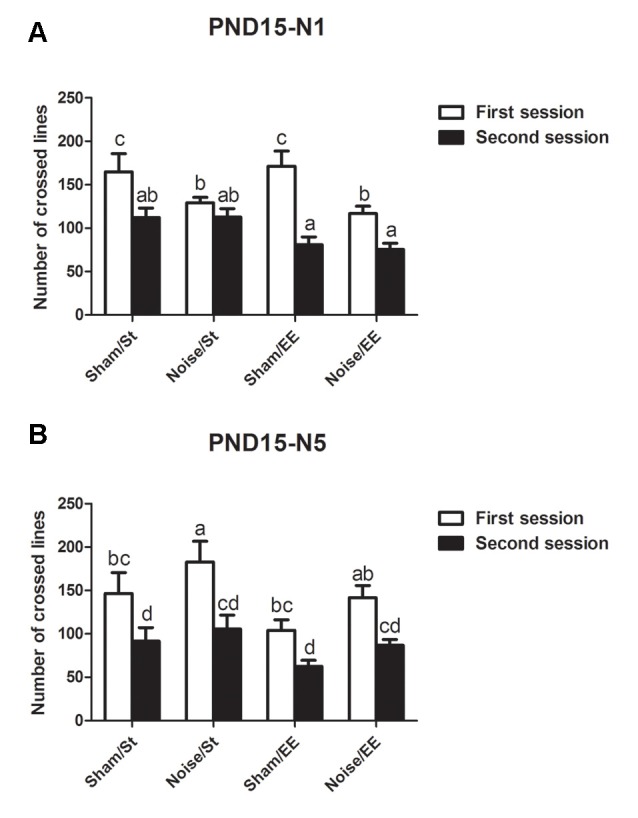
Number of lines crossed in the first and second session of the OF by PND28 animals exposed to noise at PND15. **(A)** PND15 animals exposed according to N1; **(B)** PND15 animals exposed according to N5. Sham: non-exposed animals; N1: single noise exposure; N5: five-daily noise exposure. St: standard housing. EE: enriched environment. Different letters (a, b, c, d) symbolize significant differences with *p* < 0.05. Data represent the mean ± SEM of the number of lines crossed in the first and second session of the OF, *n* = 7 for each group.

In addition, most groups showed a decrease in the lines crossed in the second session of the OF when the animals were exposed at PND15 according to N1 scheme [Three-way ANOVA, *F*_(7,47)_ = 9.65, *p* < 0.01. Between factors: exposure (sham or noise), *F*_(1,47)_ = 9.49, *p* < 0.01; housing (St or EE), *F*_(1,47)_ = 3.67, NS; within factor: session (first or second), *F*_(1,47)_ = 38.91, *p* < 0.01]. However, given that a significant interaction between exposure and session was found (*F*_(1,47)_ = 10.14, *p* < 0.01), a simple effect analysis was performed. Data show that whereas significant differences were observed between the first and second session in most groups, non-significant differences were observed in animals exposed to noise according to N1 scheme and housed in St conditions (Figure 4A, *Sham*: Two-way ANOVA, *F*_(3,21)_ = 9.34, *p* < 0.01, *post hoc* comparisons: first session vs. second session, St and EE, *p* < 0.05. *Noise*: Two-way ANOVA, *F*_(3,25)_ = 8.21, *p* < 0.01. *Post hoc* comparisons: first session vs. second session, St, NS; EE, *p* < 0.05). Finally, when animals exposed at PND15 according to N5 scheme were evaluated, significant differences were observed between the lines crossed in the first and second session of the OF in all groups [Figure 4B, *N5*: Three-way ANOVA, *F*_(7,59)_ = 5.81, *p* < 0.01. Between factors: exposure (sham or noise), *F*_(1,59)_ = 5.68, *p* < 0.05; housing (St or EE), *F*_(1,59)_ = 5.97, *p* < 0.05; within factor: session (first or second) *F*_(1,59)_ = 26.81, *p* < 0.01. *Post hoc* comparisons: first vs. second session: St (sham and noise), *p* < 0.01; EE (sham and noise), *p* < 0.05].

In summary, results show a significant decrease in the number of lines crossed in the second session of the OF when compared with the first session in most groups, both exposed at PND7 and PND15, except for animals exposed to noise at PND15 according to N1 scheme and housed in St conditions.

(ii) The number of forelimb elevations (i.e., rearing and climbing) made in the first session of the OF task was taken as an index of exploratory activity.

**Figure 5:**
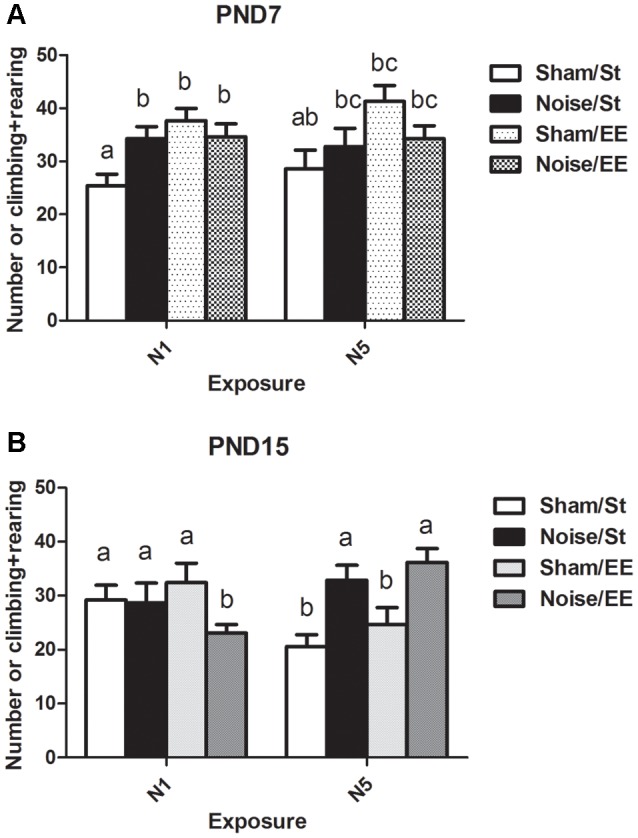
Number of elevations (climbing and rearing) made by PND28 animals exposed to noise at PND7 and PND15 in the OF task. **(A)** PND28 animals exposed at PND7; **(B)** PND28 animals exposed at PND15. Sham: non- exposed animals; N1: single noise exposure; N5: five-daily noise exposure. St: standard housing. EE: enriched environment. Different letters (a, b, c) symbolize significant differences with *p* < 0.05. Data represent the mean ± SEM of the number of elevations (climbing and rearing) made in the OF task, *n* = 7 for each group.

Data show a significant main effect in this parameter [Figure 5A, Three-way ANOVA, *F*_(7,85)_ = 3.42, *p* < 0.01. Between factors: exposure (sham or noise), *F*_(1,85)_ = 0.57, NS; housing (St or EE), *F* = 13.08, *p* < 0.01; within factors: scheme of exposure (N1 or N5) *F*_(1,85)_ = 0.55, NS]. As the interaction between exposure and housing was significant (*F*_(1,85)_ = 8.43, *p* < 0.01), a simple effect analysis was performed [*St*: Two-way ANOVA, *F*_(3,46)_ = 2.09, NS. Between factor: scheme (N1 or N5), NS; within factor: exposure (sham or noise), *p* < 0.05. *EE*: Two-way ANOVA, *F*_(3,38)_ = 1.53, NS]. The results show a significant increase in animals exposed at PND7 according to N1 and housed in St conditions when compared to their respective controls. In contrast, no changes were observed after EE housing of N1-exposed rats. Finally, exploration activity of N5-exposed animals (both after St and EE housing) remained unaltered [*post hoc* comparisons: sham vs. noise: N1: St, *p* < 0.05; EE, NS; N5 (St and EE), NS].

On the other hand, a significant main effect was found in animals exposed at PND15 (Figure 5B, Three-way ANOVA, *F*_(7,77)_ = 2.69, *p* < 0.05). As the interaction between exposure and scheme was significant (*F*_(1,77)_ = 12.65, *p* < 0.01), a simple effect analysis was performed [*N1*: Two-way ANOVA, *F*_(3,38)_ = 1.29, NS. *N5*: Two-way ANOVA, *F*_(3,37)_ = 6.35, *p* < 0.01. Between factor: exposure (sham or noise), *F*_(1,37)_ = 17.29, *p* < 0.01; within factor: scheme (St or EE), *F*_(1,37)_ = 1.74, NS, *post hoc* comparisons: sham vs. noise: N1: St, NS; EE, *p* < 0.05; N5 (St and EE), *p* < 0.05].

In summary, results show an increase in the number of forelimb elevations in animals exposed at PND7 according to N1 scheme housed in St when compared to the sham group. In contrast, no changes were observed when these animals were housed in EE or in groups exposed to N5 scheme (both after St and EE housing). On the other hand, results show that whereas no changes were observed in this parameter after exposure of PND15 animals to noise according to N1 scheme and St housing, a significant decrease was observed when exposed animals were housed in EE. In contrast, a significant increase was observed in animals repeatedly exposed to noise, both after St or EE housing.

### Elevated Plus Maze (EPM) Task

Open arms-related parameters measured in the EPM, such as the decrease in the latency to enter and an increase in the number of entries, are thought to be associated with a reduction of anxiety-like behaviors. HD in an open arm might be related with risk assessment behaviors.

#### Latency to Enter to the Open Arms in the Elevated Plus Maze (EPM) Task

**Figure 6:**
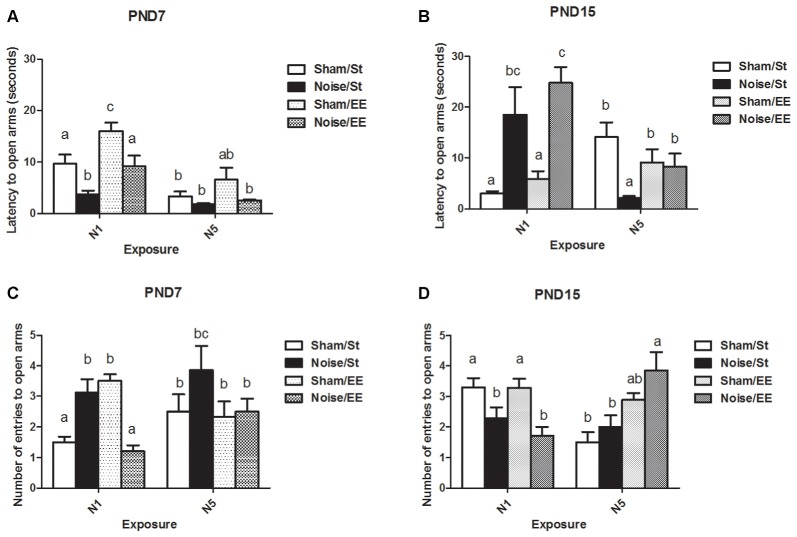
Anxiety-related behaviors measured in the elevated plus maze (EPM; latency and number of entries to open arms) of PND28 animals exposed to noise at PND7 and PND15 in the EPM task. Latency to open arms (in seconds): **(A)** PND28 animals exposed at PND7; **(B)** PND28 animals exposed at PND15. Entries to open arms: **(C)** PND28 animals exposed at PND7; **(D)** PND28 animals exposed at PND15. Sham: non- exposed animals; N1: single noise exposure; N5: five-daily noise exposure. St: standard housing. EE: enriched environment. Different letters (a, b, c) symbolize significant differences with *p* < 0.05. Data represent the mean ± SEM of latency (seconds) or entries to open arms made in the EPM task, *n* = 7 for each group.

Figure 6A shows a significant main effect on the latency to enter the open arms of the EPM when animals exposed at PND7 were evaluated [Three-way ANOVA, *F*_(7,52)_ = 10.69, *p* < 0.01; between factors: exposure (sham or noise), *F*_(1,52)_ = 23.35, *p* < 0.01; housing (St or EE), *F*_(1,52)_ = 15.60, *p* < 0.01; within factors: scheme of exposure (N1 or N5), *F*_(1,52)_ = 30.63, *p* < 0.01]. The results show a significant decrease in animals exposed to noise according to N1 scheme, both in St and EE housing conditions, without changes when exposure was done according to N5 (*post hoc* comparisons: sham vs. noise, St: N1, *p* < 0.05; N5, NS. EE: N1, *p* < 0.05; N5, NS).

When PND15 animals were exposed, a significant main effect was observed [Figure 6B, Three-way ANOVA, *F*_(7,55)_ = 5.85, *p* < 0.01; between factors: exposure (sham or noise), *F*_(1,55)_ = 5.59, *p* < 0.01; housing (St or EE), *F*_(1,55)_ = 0.45, NS; within factor: scheme (N1 or N5), *F*_(1,55)_ = 3.25, NS]. As a significant interaction was observed between exposure and scheme (*F*_(1,55)_ = 28.12, *p* < 0.01), a simple effect analysis was performed. A significant increase in the latency to open arms was found in noise-exposed animals according to N1, both in St and EE housing [Two-way ANOVA, *F*_(3,28)_ = 7.07, *p* < 0.01; between factor: exposure (sham or noise), *F*_(1,28)_ = 19.61, *p* < 0.01; within factor: housing (St or EE), *F*_(1,28)_ = 1.40, NS. *Post hoc* comparisons: sham vs. noise: St and EE, *p* < 0.05]. As a significant main effect was observed after N5 scheme [Two-way ANOVA, *F*_(3,26)_ = 4.67, *p* < 0.01; between factor: exposure (sham or noise), *F*_(1,26)_ = 8.18, *p* < 0.01; within factor: housing (St or EE), *F*_(1,26)_ = 0.03, NS] and an interaction was observed (*F*_(1,26)_ = 5.79, *p* < 0.05), a simple effect analysis was performed, which showed a significant decrease in noise-exposed animals housed in St cages when compared with their respective controls (*p* < 0.05).

In summary, results show a decrease in the latency to enter to the open arms in noise-exposed animals according to N1 at PND7 and an increase in this parameter when animals were exposed to N1 at PND15, after St and EE housing conditions. On the other hand, no changes were found when exposure was done according to N5 at PND7 whereas a significant decrease was observed when animals were exposed to N5 at PND15 and housed in St, without changes when animals were housed in EE.

#### Number of Entries to the Open Arms in the Elevated Plus Maze (EPM) Task

Figure 6C shows a significant main effect on the number of entries to the open arms of the EPM in animals exposed at PND7 (Three-way ANOVA, *F*_(7,55)_ = 4.19, *p* < 0.01). As some interactions were significant (between exposure and housing: *F*_(1,55)_ = 18.31, *p* < 0.01; between exposure, housing and scheme: *F*_(1,55)_ = 4.47, *p* < 0.05) a simple effect analysis was performed [*St*: Two-way ANOVA, *F*_(3,28)_ = 3.82, *p* < 0.05. Between factor: exposure (sham or noise), *p* < 0.01; within factor: scheme (N1 or N5), NS. *Post hoc* comparisons: sham vs. noise: St, *p* < 0.05; EE, *p* < 0.01. *EE*: Two-way ANOVA, *F*_(3,26)_ = 8.61, *p* < 0.01. Between factor: exposure (sham or noise), *p* < 0.01; within factor: scheme (N1 or N5), NS].

Data show a significant increase in St-housed animals exposed according to N1 scheme and a decrease when exposed animals were housed in EE (*post hoc* comparisons: sham vs. noise: St, *p* < 0.05; EE, *p* < 0.01). No changes were observed in rats exposed for 5 days [between factors: exposure (sham or noise) or housing (St or EE), NS; within factors: scheme of exposure (N1 or N5), NS]. However, as the interaction between exposure and scheme in rats housed within EE group was significant (*F*_(1,26)_ = 13.27, *p* < 0.01), a simple effect analysis was performed. In summary, results show a significant increase in noise-exposed animals according to N1 (*p* < 0.05) and no changes in rats exposed according to N5.

Figure 6D shows a significant main effect on the number of entries to the open arms of the EPM in animals exposed at PND15 (Three-way ANOVA, *F*_(7,60)_ = 4.77, *p* < 0.01). A decrease in this parameter was observed in animals housed in St and EE conditions and exposed according to N1 scheme. In contrast, no changes were observed in animals exposed according to N5 scheme [Between factors: exposure (sham or noise) or housing (St or EE), NS; within factors: scheme of exposure (N1 or N5), NS]. As some interactions were significant (between exposure and scheme: *F*_(1,60)_ = 12.19, *p* < 0.01; between exposure, scheme and housing: *F*_(1,60)_ = 15.21, *p* < 0.01), a simple effect analysis was performed [*St*: Two-way ANOVA, *F*_(3,30)_ = 5.15, *p* < 0.01. Between factor: exposure (sham or noise), NS; within factor: scheme (N1 or N5), *F*_(1,30)_ = 8.81, *p* < 0.01. *EE*: Two-way ANOVA, *F*_(3,29)_ = 4.85, *p* < 0.01. Between factor: exposure (sham or noise), NS. Within factor: scheme (N1 or N5), *F*_(1,29)_ = 5.83, *p* < 0.05. *Post hoc* comparisons: sham or noise: St: N1, *p* < 0.05; N5, NS. EE: N1, *p* < 0.05; N5, NS]. As a significant interaction was found between exposure and scheme, both within St and EE-housed animals (*St*: *F*_(1,30)_ = 4.63, *p* < 0.05; *EE*: *F*_(1,29)_ = 8.56, *p* < 0.01), simple effect analysis were performed. Data show a significant decrease in noise-exposed animals according to N1 scheme, both in St and EE conditions (*p* < 0.05).

In summary, results show significant differences in the number of entries to the open arms in noise-exposed animals when compared to their controls according to N1 scheme, without changes after N5 noise-exposure scheme. When animals were exposed to N1 at PND7, an increase in this parameter in St-housed animals and a decrease when animals were housed in EE were observed. Moreover, when animals were exposed at PND15 a decrease was observed, both for St and EE housing.

#### Head Dipping (HD)

**Figure 7:**
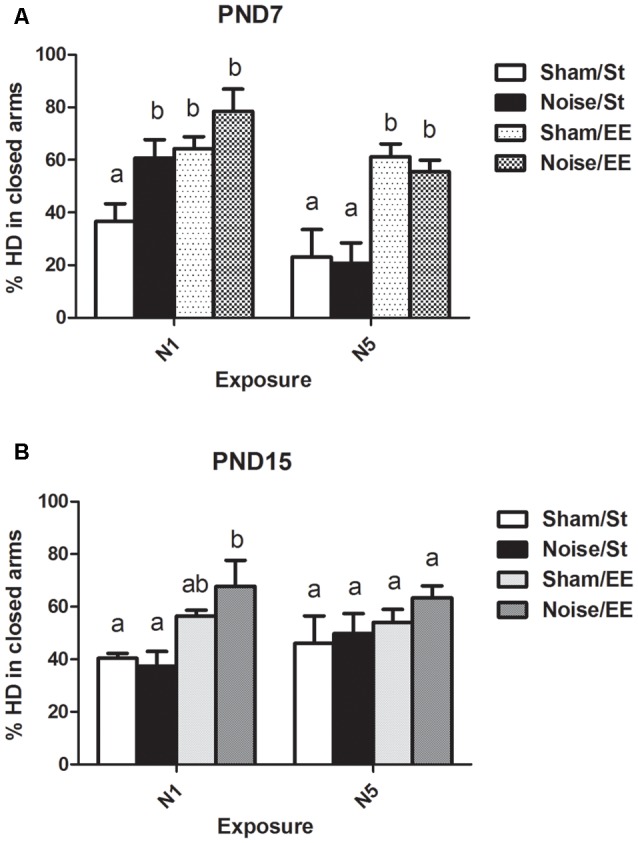
Percentage of head dipping (HD) in closed arms in the EPM task made by PND28 animals exposed to noise at PND7 and PND15. **(A)** PND28 animals exposed at PND7; **(B)** PND28 animals exposed at PND15. Sham: non- exposed animals; N1: single noise exposure; N5: five-daily noise exposure. St: standard housing. EE: enriched environment. Different letters (a, b) symbolize significant differences with *p* < 0.05. Data represent the mean ± SEM of the % of HD in closed arms made in the EPM task, *n* = 7 for each group.

When HD was analyzed, a significant main effect was observed when animals were exposed at PND7 (Figure 7A, Three-way ANOVA, *F*_(7,56)_ = 8.38, *p* < 0.01). Data show a significant increase in percentage of HD in closed (protected) arms (%HD in closed arms) between animals exposed to noise at PND7 according to N1 scheme and housed in standard conditions and sham animals, without changes when animals were housed in EE. No changes were observed when animals were exposed according to N5 scheme in comparison with the corresponding sham group [Between factors: exposure (sham or noise), NS; housing (St or EE), *F*_(1,56)_ = 36.86, *p* < 0.01; within factors: scheme of exposure (N1 or N5), *F*_(1,56)_ = 13, *p* < 0.01]. As the interaction between exposure and scheme was significant (*F*_(1,56)_ = 5.29, *p* < 0.05), a simple effect analysis was performed [*N1*: Two-way ANOVA, *F*_(3,29)_ = 6.62, *p* < 0.01. Between factor: exposure (sham or noise), *p* < 0.05. Within factor: housing (St or EE), *p* < 0.01. *N5*: Two-way ANOVA, *F*_(3,26)_ = 7.26, *p* < 0.01. Between factor: exposure (sham or noise), NS; within factor: housing (St or EE), *p* < 0.01. *Post hoc* comparisons, sham vs. noise, N1: St, *p* < 0.05; EE, NS. N5: St and EE, NS].

Finally, non-significant differences were observed between N1 and N5 noise-exposed and the corresponding sham group in PND15 animals, both in standard and EE conditions (Figure 7B, Three-way ANOVA, *F*_(7,63)_ = 1.08, NS).

In summary, results show no significant changes in %HD in closed arms in most groups, both exposed at PND7 and PND15, except from animals exposed to noise at PND7 according to N1 scheme and housed in St conditions, which showed an increase in this parameter when compared to their sham group.

### Inhibitory Avoidance (IA) Task: Ratio Between the Latency to Enter the Dark Compartment in the Retention and the Training Sessions

**Figure 8:**
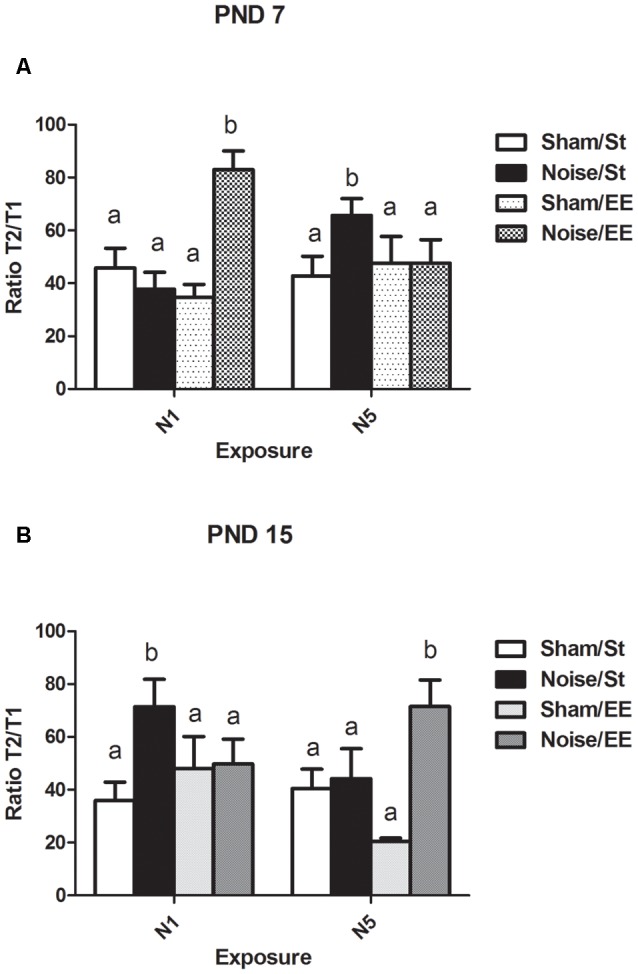
Ratio between the latency to enter the dark compartment (in seconds) in the retention session and the training session (T2/T1) in the inhibitory avoidance (IA) task in PND28 animals exposed to noise at PND7 and PND15. **(A)** PND28 animals exposed at PND7; **(B)** PND28 animals exposed at PND15. Sham: non-exposed animals; N1: single noise exposure; N5: five-daily noise exposure. St: standard housing. EE: enriched environment. Different letters (a, b) symbolize significant differences with *p* < 0.05. Data represent the mean ± SEM of T2/T1in the IA task, *n* = 7 for each group.

In the IA task, T1 is defined as the time required to enter the dark compartment (i.e., the side in which an electric shock, an aversive stimulus, was delivered) in the training session and T2 is the time required to enter the same compartment in the retention session, after an interval of 1 h. The ratio T2/T1 is the relationship between the seconds measured in the retention and the training sessions and might be taken as an index of associative memory. Figure 8A shows a significant main effect in the T2/T1 ratio in rats exposed at PND7 (Three-way ANOVA, *F*_(7,50)_ = 5.49, *p* < 0.01).Whereas non-significant differences were induced after exposure to noise under standard conditions according to N1 scheme when compared with sham animals, a significant increase was observed under EE housing [between factors: exposure (sham or noise), *F*_(1,50)_ = 9.92, *p* < 0.01; housing (St or EE), NS; within factors: scheme of exposure (N1 or N5), NS]. In contrast, an increase in this ratio was observed after repeated exposures to noise of animals housed in standard cages, without changes observed after housing in EE when compared with the corresponding sham rats. As some interactions were significant (between housing and scheme: *F*_(1,50)_ = 5.50, *p* < 0.05; between exposure, scheme and housing: *F*_(1,50)_ = 20.32, *p* < 0.01), a simple effect analysis was performed [*St*: Two-way ANOVA, *F*_(3,24)_ = 5.36 *p* < 0.01. Between factor: exposure (sham or noise), NS; within factor: scheme (N1 or N5), NS. *EE*: Two-way ANOVA, *F*_(3,25)_ = 6.85, *p* < 0.01. Between factor: exposure (sham or noise), *F*_(1,25)_ = 7.71, *p* < 0.01; within factor: scheme (N1 or N5), NS]. As the interaction between exposure and scheme was significant both in St and EE animals (*St*: *F*_(1,24)_ = 10.43, *p* < 0.01; *EE*: *F*_(1,25)_ = 10.36, *p* < 0.01), simple effect analyses were performed. *Post hoc* comparisons show a significant increase in the T2/T1 ratio of N5, St-housed, noise-exposed animals (*p* < 0.05) and in N1, EE-housed, noise-exposed animals (*p* < 0.05).

Finally, noise exposure at PND15 induced a significant main effect in the T2/T1 ratio (Figure 8B, Three-way ANOVA, *F*_(7,53)_ = 2.79, *p* < 0.01). Although a significant increase was observed in St housed animals exposed according to N1 scheme, five consecutive daily exposures did not produce changes in this parameter. Housing in an EE induced a significant increase only when rats were exposed once daily, for five consecutive days, without changes observed after N1 scheme [between factors: exposure (sham or noise), *F*_(1,53)_ = 7.84, *p* < 0.01; housing (St or EE), NS; within factors: scheme (N1 or N5), NS]. As the interaction between exposure, housing and scheme was significant (*F*_(1,53)_ = 7.89, *p* < 0.01), a simple effect analysis was performed [*St*: Two-way ANOVA, *F*_(3,26)_ = 3.30, *p* < 0.05. Between factors: exposure (sham or noise), *F*_(1,26)_ = 2.42, NS; within factor: scheme (N1 or N5), NS. *EE*: Two-way ANOVA, *F*_(3,26)_ = 3.20, *p* < 0.05. Between factors: exposure (sham or noise), *F*_(1,26)_ = 5.62, *p* < 0.05; within factor: scheme (N1 or N5), NS. *Post hoc* comparisons: sham vs. noise, EE: N1, NS; N5, *p* < 0.05]. As the interaction between exposure and scheme was significant in St animals (*F*_(1,26)_ = 4.16, *p* < 0.05), a simple effect analysis was performed. Data show a significant increase in the T2/T1 ratio of N1 St-housed, noise-exposed animals (*p* < 0.05), without changes observed in animals exposed according to N5 scheme.

In summary, data showed an increase in the ratio between the latency to enter the dark compartment in the retention and the training sessions in noise-exposed animals housed in St conditions according to N5 at PND7 and N1 at PND15, when compared with the respective controls, without changes in these groups after housing in an EE. On the other hand, an increase in noise-exposed animals was observed when compared to their sham groups, only when animals were housed in EE, according to N1 at PND7 and N5 at PND15, without changes when housed in standard cages.

### Hippocampal Trx1 and Trx2 Levels

**Figure 9:**
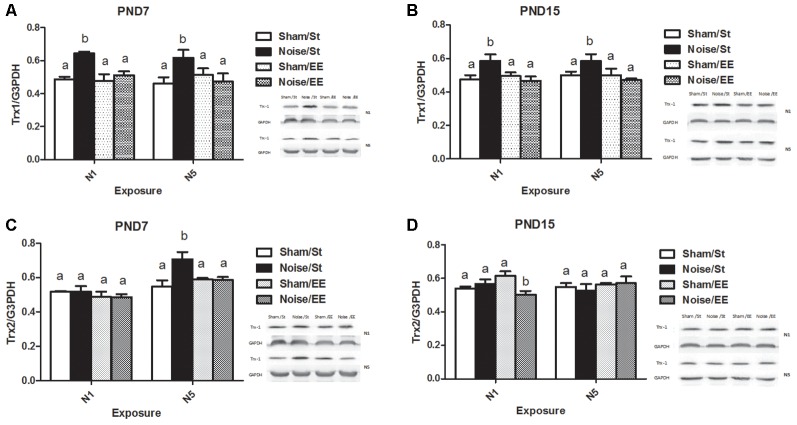
Hippocampal Thioredoxin (Trx) levels.Trx-1: **(A)** PND28 animals exposed at PND7; **(B)** PND28 animals exposed at PND15. Trx-2: **(C)** PND28 animals exposed at PND7; **(D)** PND28 animals exposed at PND15. Sham: non- exposed animals; N1: single noise exposure; N5: five-daily noise exposure. St: standard housing. EE: enriched environment. Different letters (a, b) symbolize significant differences with *p* < 0.05. Data represent the mean ± SEM of the levels of hippocampal Trx-1 or Trx-2, *n* = 4 for each group.

Figure 9A shows that noise exposure at PND7 induced a significant increase in hippocampal Trx-1 levels, when exposed according to both N1 or N5 schemes [Three-way ANOVA, *F*_(7,42)_ = 2.82, *p* < 0.05. Between factors: exposure (sham or noise), *F*_(1,42)_ = 6.08, *p* < 0.05; housing (St or EE), *F*_(1,42)_ = 4.17, *p* < 0.05; within factors: scheme (N1 or N5), NS], that remained similar to the corresponding sham levels when animals were housed in an EE. As interaction between exposure and housing was significant (*F*_(1,42)_ = 8.32, *p* < 0.01), a simple effect analysis was performed [*St*: Two-way ANOVA, *F*_(3,20)_ = 5.47, *p* < 0.01. Between factor: exposure (sham or noise), *F*_(1,20)_ = 16.05, *p* < 0.01; within factor: scheme (N1 or N5), NS. *EE*: Two-way ANOVA, *F*_(3,21)_ = 0.27, NS. Between factor: exposure (sham or noise), NS; within factor: scheme (N1 or N5), NS. *Post hoc* comparisons: St: sham vs. noise: N1 and N5, *p* < 0.05. EE: N1 and N5, NS].

Similarly, animals exposed to noise at PND15 showed a significant increase, according to N1 or N5 schemes and housed in St conditions, that remained similar to the corresponding sham values when housed in EE [Figure 9B, Three-way ANOVA, *F*_(7,36)_ = 2.67, *p* < 0.05. Between factors: exposure (sham or noise), *F*_(1,36)_ = 4.18, *p* < 0.05; housing (St or EE), *F*_(1,36)_ = 4.76, *p* < 0.05; within factors: scheme (N1 or N5), NS]. As interaction between exposure and housing was significant (*F*_(1,36)_ = 9.72, *p* < 0.01), a simple effect analysis was performed [*St*: Two-way ANOVA, *F*_(3,19)_ = 3.47, *p* < 0.05. Between factor: exposure (sham or noise), *F*_(1,19)_ = 10.41, *p* < 0.01; within factor: scheme (N1 or N5), NS. *EE*: Two-way ANOVA, *F*_(3,16)_ = 0.40, NS. Between factor: exposure (sham or noise), NS; within factor: scheme (N1 or N5), NS. *Post hoc* comparisons: sham vs. noise: St: N1 and N5, *p* < 0.05. EE: N1 and N5, NS].

Figure 9C shows a significant main effect on Trx-2 in animals exposed at PND7, although significant differences between sham and noise-exposed animals were observed only after five repeated exposures in standard housing [Three-way ANOVA, *F*_(7,36)_ = 6.05, *p* < 0.01. Between factors: exposure (sham or noise), *F*_(1,36)_ = 3.17, NS; housing (St or EE), *F*_(1,36)_ = 1.80, NS; within factors: scheme (N1 or N5), *F*_(1,36)_ = 28.17, *p* < 0.01. *Post hoc* comparisons: sham vs. noise: *N1*: St and EE, NS; *N5*: St, *p* < 0.05; EE, NS]. In contrast, non-significant main effects were observed in animals exposed at PND15 (Figure 9D, Three-way ANOVA, *F*_(7,34)_ = 1.34, NS). However, as a significant interaction was found between exposure, housing and scheme (*F*_(1,34)_ = 4.43, *p* < 0.05), a simple effect analysis was performed. In addition, although no changes were induced in rats housed both in St and EE conditions (Two-way ANOVA, St: *F*_(3,17)_ = 0.40, NS; EE: *F*_(3,16)_ = 2.47, NS), a significant interaction was found between exposure and scheme in rats housed in EE (*F*_(1,16)_ = 4.42, *p* < 0.05), with a significant decrease only in rats exposed according to N1 scheme (*p* < 0.05).

In summary, results show an increase in hippocampal Trx1 levels in all noise-exposed animals when compared to their respective sham groups, according to both schemes (N1 and N5) and ages of exposure (PND7 and PND15), when animals were housed in St condition, without changes after EE housing. Moreover, data show an increase in hippocampal Trx2 levels in PND7 noise-exposed animals only after five repeated exposures in standard housing when compared to their sham group, without changes after EE housing. Finally, although no changes in Hippocampal Trx2 levels were induced in rats exposed to noise according to N1 scheme and housed both in St and EE conditions at PND15, a significant decrease was observed in animals exposed to noise according to N1 and housed in EE when compared to the sham group.

## Discussion

Present results show that exposure of 7 and 15-days-old animals to moderate levels of white noise (95–97 dB SPL, 2 h), using single or repeated session’s exposures, was capable to trigger hippocampal-related behavioral alterations as well as oxidative-related molecular changes when evaluated after several days, that differed according to the scheme used. In addition, animals were not uniformly affected when different ages of exposure were compared. The housing in an enriched environment, a non-pharmacological strategy of neuroprotection, was effective in preventing some of these changes that differed between the different groups. Finally, non-significant changes in auditory function were found in neither group.

### Auditory Pathway Evaluation

No changes in the auditory thresholds were induced, neither when the rats were exposed at PND7 nor at PND15, supporting results in other animal models (Pienkowski and Eggermont, 2012; Gourévitch et al., 2014). The fact that auditory system become mature at approximately PND12, could explain why there were no significant changes in the auditory threshold of animals exposed at PND7, considering that auditory pathway was not functional at the age of exposure. For this reason, the observation of damage when exposure was done at PND7, an age at which the auditory pathway is still immature, might suggest that moderate noise exposure can produce the behavioral and biochemical effects through a direct rather than an indirect mechanism, as hypothesized by Säljö et al. (2011). Otherwise, it is possible that in the case of rats exposed at PND15, which already had a functional auditory pathway, the intensity of noise used was not high enough to generate an effect on the auditory thresholds at PND28. However, it should not be discarded that animals’ auditory system could be affected after PND 12 and prior to PND28, age at which animals were evaluated.

### Behavioral Assessment

The behavioral alterations found in PND15 animals exposed according to N5 scheme differed from those observed in PND7 rats subjected to the same noise scheme, as was previously found for animals exposed at PND7 and PND15 according to N1 scheme (Uran et al., 2010, 2012, 2014; Molina et al., 2016a). Even more, when exposed animals were housed in an EE, prevention of most behavioral alterations was observed in all groups. These data suggest that a prompt housing intervention, soon after single or multiple exposures to an environmental potentially hazardous agent, could be effective to avoid unfavorable effects, mainly if it is implemented in early stages of development (Smith et al., 2018; Gong et al., 2018).

It is important to highlight that habituation memory refers to behavioral changes that could be triggered in response to repeated exposure to novelty (Leussis and Bolivar, 2006). In addition, fear conditioning (i.e., inhibitory avoidance) implies a predictive relationship between a stimulus and an event (Ennaceur and Delacour, 1988). Interestingly, both depend on the hippocampal integrity (Vianna et al., 2000; Leussis and Bolivar, 2006). Finally, exploration is a behavior that can be measured in the OF and is triggered by novel stimuli: consists of behavioral acts and postures that permit an animal to collect information about new aspects of the environment (Barros et al., 2006). However, there are some debate in the literature (Ennaceur, 2014), as several authors suggested that as the anxiety-like behavior decreases, the animals increase the exploration of the environment (Escorihuela et al., 1999; Prut and Belzung, 2003; Lever et al., 2006; Kalouda and Pitsikas, 2015), whereas others postulated that it may be interpreted as an anxiogenic-like behavior (Barnett and Cowan, 1976; Lamprea et al., 2008).

#### Habituation Memory

When a rodent is placed in a novel environment, it begins to form an internal representation of the surrounding spatial information. Once this hippocampal-dependent map is “complete,” the animal decreases the exploration of the environment because it would be considered habituated to the new context (O’Keefe and Nadel, 1979; Leussis and Andersen, 2008). Given that impairment in this parameter was observed only in PND15N1 animals and considering that the deficit was not evident when younger animals were exposed to noise, habituation memory might be used as a marker of vulnerability. Therefore, as the auditory system becomes active between PND7 and PND15 (de Villers-Sidani et al., 2008; Säljö et al., 2011), it could be postulated that more immature animals could be refractory to the damaging effects of noise on this type of memory, probably due to the impossibility of noise to affect CNS by means of a functional auditory system. As no effect was observed when PND15 animals were exposed to noise for 5 days, it could be suggested that repeated exposures might trigger adaptive mechanisms intended to counteract potential damage (Febbraro et al., 2017; Scott et al., 2017). The ability of EE to prevent noise-induced changes in PND15N1 animals might depend on the same adaptive mechanisms.

#### Exploratory Activity

Significant differences among groups were observed in exploratory activity, with an increase in those exposed to noise at PND7N1 and PND15N5 and without changes in the other groups. As a decrease in the latency and/or an increase in the number of entrances to open arms of the EPM was also observed in both groups, it could be suggested that greater exploration might be associated with decreased anxiety-like behavior, supporting Kalouda and Pitsikas (2015) and Wright et al.’s (2011) results. In addition, it could be claimed that an increase in exploratory activity with the consequent collection of information from the environment can favor the habituation and adaptability of these animals. Furthermore, an increase in novelty anxiety triggered by the new environment might affect exploration and habituation (Leussis and Bolivar, 2006), because shared mechanisms might be involved (Izquierdo and Medina, 1997; Salomons et al., 2012).

Conversely, animals with impairment in habituation memory (i.e., those exposed at PND15N1) did not exhibit changes exploratory activity. Even more, the increase in anxiety-related observed in animals exposed at PND15N1 might be related to a deficit in habituation memory (Venero et al., 2005; Barzegar et al., 2015). Furthermore, rats exposed at PND15N1 could have an increased fear response, which would imply that these animals would have greater emotional reactivity.

However, whereas housing in an EE prevented the changes in exploration, as observed in PND7N1 rats, no prevention was observed when animals exposed at PND15N5 were evaluated. These data suggest that there would seem to be a window of opportunity to intervene using a neuroprotection strategy that depends on the developmental stage at which the injury took place (Smith et al., 2018; Gong et al., 2018).

#### Emotional Reactivity: Anxiety-Like Behavior and Risk Assessment Behavior

It should be considered that decreased anxiety-like behavior could be interpreted as a behavioral improvement. However, it could not be true in the wild, because certain minimal anxiety levels might be required to cope with eventual dangerous situations. In contrast, although low or moderate levels of anxiety may be positive for learning and memory processes, it has been shown that high levels could lead to a cognitive deficit (Silva and Brandão, 2000).

A decrease in the entries to open arms of the EPM might be taken as a sign of an increase in anxiety-like behavior, as observed in animals exposed at PND15N1 and supported by Angrini and Leslie (2012). Conversely, an increase in the entries might imply a decrease in anxiety-like behavior, as observed in PND7N1 and supported by Eraslan et al. (2015). Therefore, it could be suggested that not only the developmental stage at which the animals are exposed to the environmental agent but also the scheme of exposure come into play to determine the development of emotional alterations. The lack of change of anxiety-like behavior in animals subjected to five daily noise sessions (PND7N5 and PND15N5) could be explained by a possible compensation that could be triggered as a consequence of the repeated exposure to the environmental challenge.

%HD in closed arms is a significant behavioral dimension whose biological function is to inform behavioral strategies in potentially dangerous situations (Carobrez and Bertoglio, 2005). Noise was capable to increase this parameter only when animals were exposed at PND7N1. Actually, animals with decreased anxiety-like behaviors would be less cautious and could be more exposed to potential hazards. As a decrease in anxiety-like behaviors was observed in the group exposed at PND7N1, the finding of an increase in risk assessment behavior might not support this hypothesis. This result implies that at an early developmental age noise exposure increased the consciousness against potential dangers, such as the open environment of the OF task (Rodgers and Cole, 1993). Neither repeated exposure sessions nor maturation was able to induce changes, suggesting that this unique defensive behavior in mammals that reduces the chances of the animal to being harmed might be more important in helpless animals and tend to disappear with the advancement of CNS maturation. In contrast, the increases in this behavior observed in PND7N1 animals can be effectively prevented by housing in EE, suggesting that animals exposed to noise at earlier ages could be handled through the modification of rearing conditions when subjected to a threatening situation.

Interestingly, EE has shown “*per se*” to increase %HD when non-exposed PND7N1 rats were tested, when compared with the respective groups housed in standard conditions, indicating that these ethological readings might be altered through an environmental intervention, supporting results of Pietropaolo et al. (2004a) using a mice model of housing in an enriched environment. Usually, an increase in this risk assessment behavior is correlated with a decrease in anxiety-like behaviors (Cole and Rodgers, 1995). In contrast, non-exposed PND15 animals housed in EE cages showed unchanged %HD when compared with those animals housed under standard conditions, suggesting that the age of exposure is critical to driving this emotional output.

#### Associative Memory

Associative memory can be evaluated through the IA task by means of the ratio between the seconds taken to enter the dark compartment in the retention and the training sessions (T2/T1, Roozendaal, 2002). Although all animals retained associative memory in this task, the performance in the associative memory task was increased in rats exposed at PND7N5 and PND15N1, suggesting that these animals would have a more detailed representation of the traumatic event, as reported by Atucha and Roozendaal (2015). Again, the lack of change in the other groups might be related to either immature associative mechanisms (PND7N1) or to adaptive mechanisms (PND15N5) that could be triggered by repeated exposures, intended to counteract potential damage, as observed in different stress models (Febbraro et al., 2017; Scott et al., 2017). However, as memory retention has been observed in both groups, it should not be discarded that PND7N5 and PND15N1 rats experimented an increase in fear sense instead of an improvement in associative memory (i.e., there seems not to be a memory acquisition trouble). It must be underlined that fear can be distinguished from anxiety as it occurs in response to threats perceived as imminent, while anxiety could occur in response to potential or sustained threats (Izquierdo et al., 2016). In other words, a greater fear sense that could explain the increase in T2 in the IA task could be distinguished from the anxiety in response to a potential danger that occurs in the EPM test. There is also evidence supporting this statement, demonstrating that anxiety and fear response could depend on different CNS structures (Kjelstrup et al., 2002; Pentkowski et al., 2006).

In consequence, it could be suggested that the longer latency to enter into the dark compartment might be related to an increased emotional reactivity, a non-adaptive response, as suggested by Costanzi et al. (2011) that might be also related to the increase in anxiety-related behavior, as observed in humans (Michael et al., 2007; Ponomarev et al., 2010). Therefore, although fear is essential for survival, destined to learn about a potential danger, the lack of behavioral flexibility might expose individuals to environmental changes that might affect not only hippocampus but also other structures-related behaviors (Barros et al., 2000; Izquierdo et al., 2016).

In addition, whereas EE was able to prevent the noise-induced changes in the associative memory of PND7N5 and PND15N1 groups, this housing condition induced an improvement in the performance of noise-exposed PND7N1 and PND15N5 groups, suggesting that differences in environmental stimulation could favor different behavioral phenotypes in the presence of an unfavorable previous condition, such as exposure to noise.

### EE as a Neuroprotective Strategy

The EE has shown to be an effective tool to protect against CNS injury (Lores-Arnaiz et al., 2006), obtaining benefits on learning and memory (Schrijver et al., 2002; Baraldi et al., 2013) as well as on anxiety-like behaviors (Friske and Gammie, 2005; Lima et al., 2014).

It should be highlighted that short periods of housing in an enriched environment appeared to be enough to produce brain changes in young, but not in adults rats, suggesting that in rodent species adolescence is a highly sensitive period likely to be modified by environmental challenges (Spear, 2000). Actually, only 1 week of EE used in the present experimental model as a neuroprotective strategy contrasts with the long periods required to be protective when adult animals are the experimental subjects, supporting this hypothesis.

In addition, housing in EE generated changes on its own in some behavioral parameters. For example, behavioral differences were observed between control groups depending on the type of housing in parameters such as anxiety-like behavior, %HD and exploratory activity. In addition, in some cases, exposed animals presented changes in their behavior when compared with their respective sham group only when they were housed in an EE, whereas no differences were observed when housed under standard conditions. Supporting these observations, several authors found behavioral changes in untreated animals after housing in EE, even during short periods (van Praag et al., 1999; Nithianantharajah and Hannan, 2006; Mitra and Sapolsky, 2012; Sampedro-Piquero and Begega, 2017). It has been postulated that beneficial effects of EE could be due to the novelty and increased social contact and exercise, which are rewarding for animals as well as efficacious in supplying for their ethological needs (Pietropaolo et al., 2004b; Crofton et al., 2015).

Therefore, it could be suggested that housing for a week in an EE was able to generate behavioral changes by itself, as well as to unmask differences between exposed animals and their controls, which highlight the importance of the interaction with the environment that surrounds the animal, given that differences in environmental stimulation may favor the development of certain behavioral phenotypes (Nithianantharajah and Hannan, 2006; Mychasiuk et al., 2012).

### Is Hippocampal Oxidative State Involved?

Finally, it is known that the balance of cellular oxidative status might be affected after several insults (Bendix et al., 2012; Sies, 2015). A significant increase in the antioxidant enzymes activities might indicate that a prior increase in ROS production could have been triggered, suggesting that the brain endogenous antioxidant defense system is capable of being activated in response to excessive ROS generation. As some changes in hippocampal oxidative status were observed after noise exposure of PND15N1 animals (Uran et al., 2010, 2014), the measurement of Trx, an endogenous antioxidant often involved in brain injuries, could be taken as a marker of damage in the present model that could underlie behavioral changes. However, although a similar increase in hippocampal Trx-1 levels was found in all groups, dissimilar changes in the behavioral parameters in each group were observed. This lack of correlation suggests that this endogenous antioxidant could not be the main responsible for the behavioral changes. Although, Trxs are a part of the vast antioxidant machinery, these key enzymes are frequently altered in oxidative-related pathologies. Nevertheless, other markers should be measured to confirm these findings.

## Conclusion

In conclusion, noise exposure using single or repeated session’s schemes was capable to trigger hippocampal-related behavioral alterations as well as oxidative-related molecular changes in animals exposed at PND 7 and PND15 and evaluated after several days that differed according to the scheme used and the age of exposure. Housing in an enriched environment, a non-pharmacological strategy of neuroprotection, was effective in preventing some of these changes. In addition, an oxidative imbalance might be triggered in the hippocampus of rats from all groups, without changes in the auditory function.

The different ages of exposure, as well as the different schemes applied, might predispose animals to undergo different alterations: more behavioral alterations were observed in younger animals, exposed for a single day. Therefore, it could be suggested that immature animals might be more vulnerable to noise impact and that the alterations induced by repeated exposures might be more effectively compensated in younger animals.

Therefore, these findings suggest that after repeated exposure to an environmental challenge animals become less susceptible to noise-induced behavioral changes, probably due to the ability of adaptation to an unfavorable condition. Moreover, it could be hypothesized that damage to younger individuals could be more easily prevented by an environmental manipulation.

The knowledge of the mechanisms involved in the damage, as well as the strategies aimed to prevent them, is of clinical relevance considering noise exposure as a public health problem that is increasing in urbanized societies.

## Data Availability

The datasets generated for this study are available on request to the corresponding author.

## Ethics Statement

This study was carried out in accordance with the Institutional Committee for the Use and Care of Laboratory Animal rules (CICUAL, School of Medicine, University of Buenos Aires, Argentina). The present experimental protocol was approved by this Committee and registered with the number 53679/16. The CICUAL adheres to the rules of the “Guide for the Care and Use of Laboratory Animals” (NIH; 2011 revision) and to the EC Directive 86/609/EEC (2010 revision) for animal experiments.

## Author Contributions

SM performed the experiments, with the collaboration of GB and MR. MG-C performed auditory measurements. LG wrote the manuscript, with the collaboration of FC.

## Conflict of Interest Statement

The authors declare that the research was conducted in the absence of any commercial or financial relationships that could be construed as a potential conflict of interest.

## Abbreviations

HC: Hippocampus
PND7: Rats exposed to noise at 7 days of age
PND15: Rats exposed to noise at 15 days of age
N1/N5: Exposure schemes: 1 day and 5 days, respectively
St: Standard cage
EE: Enriched environment
Trx-1, Trx-2: Thioredoxin-1 and Thioredoxin-2, respectively
CNS: Central Nervous System.
